# Cerebellar Hemangioblastoma Associated With a Vascular Malformation: A Case Report Treated With Surgery and Radiosurgery

**DOI:** 10.7759/cureus.16549

**Published:** 2021-07-21

**Authors:** Yasuhiro Matsushita, Yoshihisa Kida, Yoshimasa Mori

**Affiliations:** 1 Neurological Surgery, Gamma Knife Center, Ookuma Hospital, Nagoya, JPN; 2 Neurological Surgery, Ookuma Hospital, Nagoya, JPN; 3 Radiology and Radiation Oncology, Aichi Medical University, Nagakute, JPN; 4 Neurological Surgery, Aoyama General Hospital, Toyokawa, JPN; 5 Radiation Oncology and Neurological Surgery, Shin-Yurigaoka General Hospital, Kawasaki, JPN

**Keywords:** hemangioblastoma, arteriovenous malformation, cerebellum, brain, radiosurgery, gamma knife, arteriovenous fistula

## Abstract

Hemangioblastoma is well known as an essentially benign cystic and/or solid tumor classified WHO grade I, mainly originated in the posterior fossa. One of the characteristics of this tumor is very rich vasculature in and around the tumor. We have encountered a case of hemangioblastoma in association with a vascular anomaly near the tumor, though they were located separately by the tentorium. A vascular anomaly with arteriovenous (AV) shunting flow in the left occipital lobe was verified at angiography, which received a blood supply from left occipital artery and drained to occipital cortical veins. Successful removal of the cerebellar tumor and pathological diagnosis of hemangioblastoma was made. The second angiography in our hospital demonstrated the same vascular anomaly above the tentorium with feeding artery of posterior cerebral artery, a small nidus, and cortical draining veins, which were less obviously stained. Only the arteriovenous malformation (AVM) nidus in sigmoid vein was targeted for radiosurgery and 20 Gy at the margin was delivered. Since AV shunting was less remarkable on the second angiography than that on the first angiography may be because of a decreased vascular supply to the supratentorial AVM after surgical resection of the infratentorial hemangioblastoma and might indicate an indirect connection between the two lesions.

## Introduction

Hemangioblastoma is well known as an essentially benign cystic and/or solid tumor classified as WHO grade I, mainly originated in the posterior fossa. One of the characteristics of this tumor is very rich vasculature in and around the tumor. Since they are considered to be benign, surgical extirpation is the first treatment choice. Because of rich vascularity, endovascular embolization is often required before surgery. During the surgery, the tumor may occasionally disseminate around or apart from the original site. Treatment with radiosurgery was often employed with a favorable tumor control for residual tumors. A few cases have been reported who developed hemangioblastoma in association with vascular anomalies [1-7]. We have encountered a case of hemangioblastoma associated with an arteriovenous malformation (AVM) near the tumor, though they were located separately below and above the tentorium. The hemangioblastoma was successfully resected totally and the AVM nidus was treated by Gamma Knife radiosurgery. Our experience of a co-existence of hemangioblastoma and vascular anomaly will be reported.

## Case presentation

**Figure 1:**
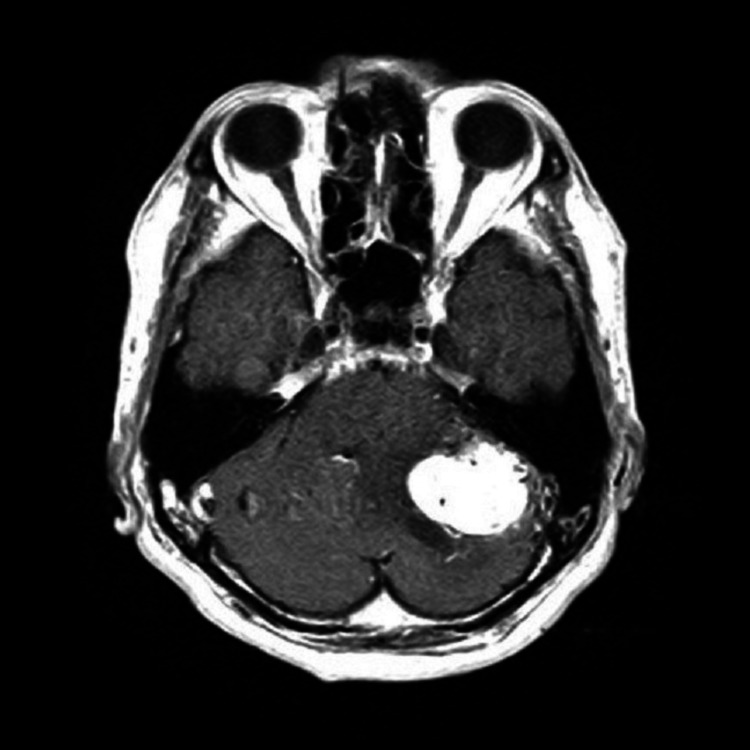
Magnetic resonance imaging (MRI) on admission. Gadolinium (Gd)-enhanced T1-weighted  MRI showed a large tumor in the left cerebello-pontine angle.

A 70-year-old gentleman suffering from a persistent headache, visited a nearby hospital. His neurological studies demonstrated no special neurological signs. However, his radiological study with magnetic resonance imaging (MRI) showed a large tumor associated with small cyst in the left cerebellum causing apparent hydrocephalus (Figure 1).

**Figure 2:**
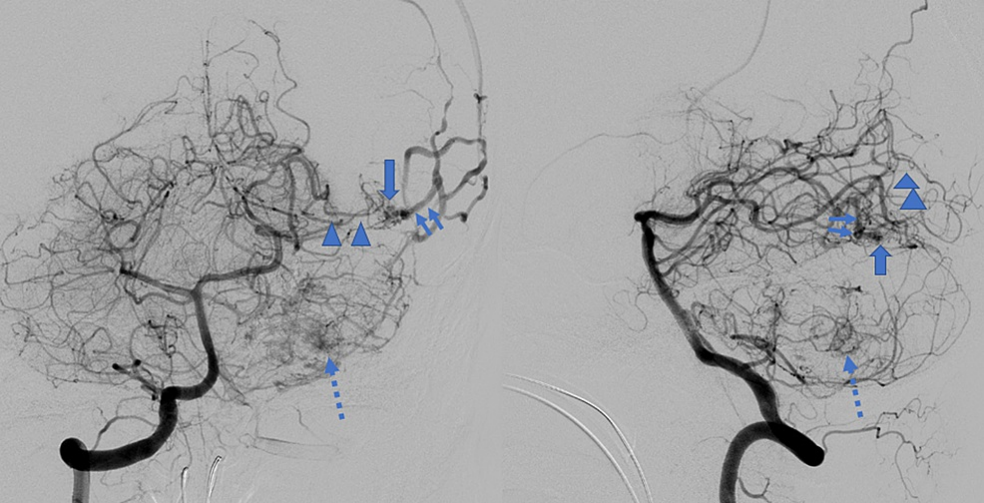
Preoperative angiography (anteroposterior [AP] view, before embolization) Preoperative angiography demonstrated an apparent tumor stain in cerebello-pontine angle (dotted arrow). In addition, a vascular malformation with arterio-venous (AV) shunting was also visualized just on the tentorium, whose feeding artery was posterior cerebral artery and drainers were occipital cortical veins.

Preoperative angiography demonstrated tumor stain in association with the infratentorial tumor. In addition, an atreriovenous malformation (AVM) in the supratentorial portion (Figure 2).

**Figure 3:**
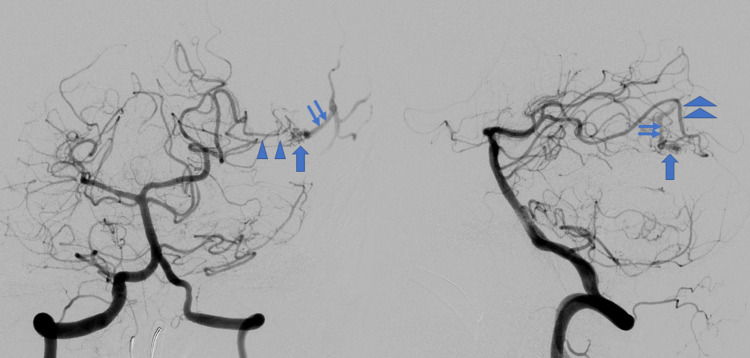
Angiography after preoperative embolization. AP view (left) and lateral one (right). After preoperative embolization, the tumor stains were far decreased. The arteriovenous malformation (AVM) was visualized almost the same as at pre-embolization. Arterio-venous shunting was confirmed consisting of left posterior cerebral artery (arrowheads), a nidus (thick arrow), and draining cortical veins (thin arrows).

Presurgical embolization of the tumor vessels was performed (Figure 3). An AVM nidus in the left occipital lobe was still verified on angiograms, which received a blood supply from left posterior cerebral artery and drained into occipital cortical veins (Figure 3).

The cerebellar tumor was totally resected and histological diagnosis of hemangioblastoma was verified. After the surgery, he showed a full recovery without any neurological deficits. No bruit was audible on the left mastoid bone. He was referred to our Radiosurgery Center in order to treat the AVM a month later.

**Figure 4:**
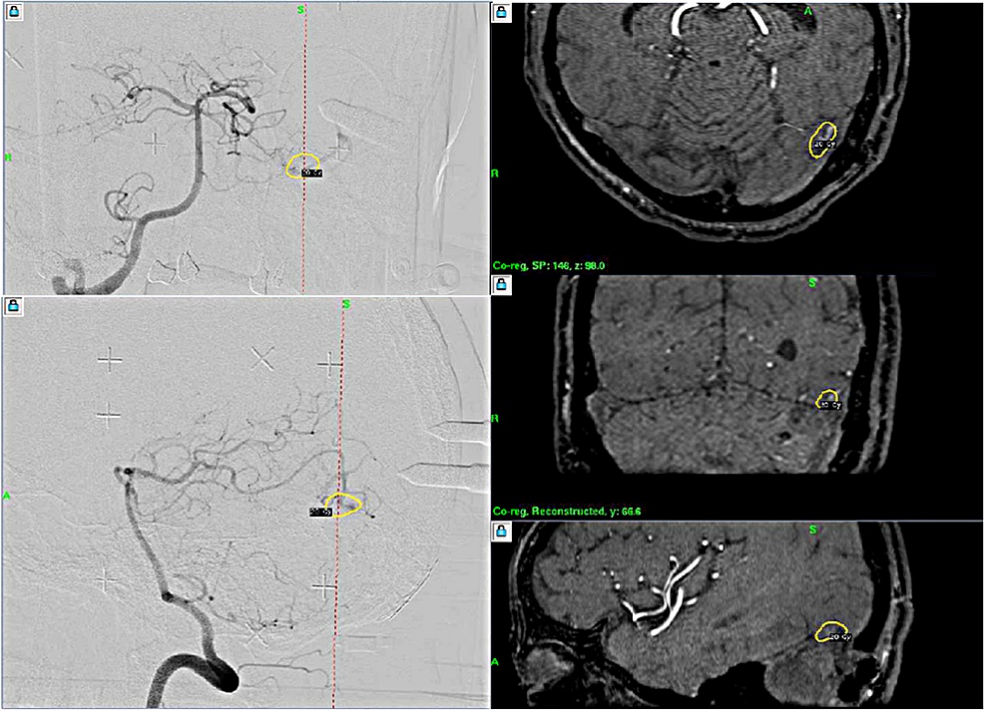
Gamma Knife radiosurgery for arteriovenous malformation. The nidus of arteriovenous malformation (AVM) just above the tentorium was treated by stereotactic radiosurgery with a marginal dose of 20 Gy. Shunting flow of the AVM looked less remarkable when compared with the preoperative angiography.

The second angiography just before Gamma Knife radiosurgery in our hospital demonstrated the same vascular anomaly above the tentorium with feeding artery of posterior cerebral artery, AVM nidus, and cortical draining veins. The arteriovenous shunt flow looked less obviously stained than that on the initial angiography performed before surgical resection of hemangioblastoma. Only the AVM nidus just above the tentorium was targeted for radiosurgery and a marginal dose of 20 Gy was delivered (Figure 4).

There were no troubles during the Gamma Knife procedure and he remained with no neurological deficits. Follow-up studies are scheduled for several months later.

## Discussion

Although a rich vascular supply, mural nodule and cyst formation are frequently seen in the tumor of hemangioblastoma, other vascular anomalies near or adjacent to the tumor are not frequent. Several reports have been published as case studies in the literature [1-4,8-10] (Table 1).

**Table 1:** Reported case of hemangioblastoma with vascular anomaly. M: male; F: female; AVM: arteriovenous malformation; AICA: anterior inferior cerebellar artery; PICA: posterior inferior cerebellar artery; CPA: cerebellopontine angle.

Study(year)	Age/sex	Relationship of hemangioblastoma and vascular anomaly	Location		Presentation	Treatments	
			Hemangioblastoma	Vascular anomaly		Hemangioblastoma	Vascular anomaly
Raynor and Kingman (1965) [6]	19/M	Intermixed	Cerebellar-vermian	Large midline vascular lesion, suggestive of AVM	Hemorrhage of lesion (AVM?)	Resection	Resection
Thaggard et al. (1977) [7]	38/F	Intermixed	Cerebellar-right cerebellar hemisphere	The cerebellar midline with multiple feeding arteries and draining veins	Symptomatic of increased intracranial pressure (tumor?)	Resection	Resection
Medvedev et al. (1991) [3]	38/M	Intermixed	Cerebellar–mesobasal aspect of left cerebellar hemisphere	Left AICA and PICA feeder vessels rapidly shunting to the tentorial sinuses	Symptomatic hydrocephalus and cerebellar signs (tumor)	Resection	Resection
Monserrate et al. (2020) [2]	40/M	Intermixed	cerebellar-right cerebellar hemisphere.	Right cerebellar meningeal Artery and PICA feeder vessels	Hemorrhage of lesion	Resection	Resection
Bennett et al. (2016) [5]	44/F	Separate (place and time)	Cerebellar-vermian	Left SCA and PICA feeder vessels	Symptomatic hydrocephalus and cerebellar signs(tumor). One year later hemorrhage of lesion (AVM)	Resection	Resection
Healy et al. (2020) [1]	46/M	Intermixed	Cerebellar and spinal–vermian and including posterior cervical cord	No angiographic phase pre-operatively	Symptomatic hydrocephalus and cerebellar signs	Resection	Resection
Ours (2021)	70/M	Separate (place)	Cerebellar-left CPA	Left occipital artery feeder vessels	Symptomatic hydrocephalus and cerebellar signs (tumor)	Resection	Radiosurgery

Although two disease entities were incidentally found, the majority of them were found near the tumors. Some others were identified after the surgery of the hemangioblastoma in the tumor site. In the latter, a certain possible relation of the two has to be considered and remained tumor vessels with AV shunting might be possible. Bennett et al. [5] suggested that AVM blood flow may increase over time. In their report, no AVM was observed during preoperative MRI or tumor resection. Follow-up MRIs at six and 12 months showed a slight increase in vascular distribution that was not present on first MRI, suggesting that AVM may have developed or matured after hemangioblastoma resection. Removal of the hemangioblastoma could have altered flow dynamics in an interrelated AVM and caused an asymptomatic vascular lesion to mature into a clinically symptomatic AVM.

 In our case, the tumor and the vascular anomaly were near in location, but both were separated by tentorium and had no direct connection. A disseminated tumor formation is not probable, therefore appearance of the two lesions is seemingly incidental. However, AV shunting was less remarkable on second angiography after surgical resection of the hemangioblastoma which may indicate a decreased blood supply to the vascular anomaly and might have indirect connection between the two.

The treatment choice after the surgery seems to be controversial. However, it seems to be adequate to perform radiosurgery, because the abnormal vascular anomaly remained with an apparent cortical reflux. Cortical reflux may have danger of bleeding in and around the occipital lobe. Since hemangioblastomas are known to be not radioresistant, radiosurgery is one of the options [8-10]. In our case, hemangioblastoma and AVM are located just below and above the tentorium, it might be possible to treat two pathologies by single session of radiosurgery. However, our treatment strategy is adequate since the hemangioblastoma is large in volume and not ideal for radiosurgery.

## Conclusions

A case of cerebellar hemangioblastoma combined with AVM was reported. The lesions were treated by first embolization of tumor vessels, and the microsurgery surgery and at the last with radiosurgery for AVM without any adverse effects. Since various abnormal vessels may accompany in and around hemangioblastoma, vascular anomalies such as AVM, AVF, aneurysm may accompany adjacent to or near the tumors. For these cases, definite strategies including microsurgery, endovascular treatment as well as radiosurgery may be required. In conclusion, sufficient care should be made not only for the diagnosis, but also for the treatment of posterior fossa hemangioblastomas, since the development of vascular anomaly may accompany. In all the reported cases, vascular anomalies are located near or adjacent to the tumors. Therefore the relations between the two are not incidental and altered hemodynamic changes may or might be involved in this coexistence.
